# *N*-[(*Z*)-(1-Methyl-1*H*-pyrrol-2-yl)methyl­idene]-1*H*-1,2,4-triazol-5-amine

**DOI:** 10.1107/S160053680804097X

**Published:** 2008-12-10

**Authors:** Zahid H. Chohan, Muhammad Hanif, M. Nawaz Tahir

**Affiliations:** a Department of Chemistry, Bahauddin Zakariya University, Multan 60800, Pakistan; b Department of Physics, University of Sargodha, Sargodha, Pakistan

## Abstract

In the title compound, C_8_H_9_N_5_, a Schiff base derived from *N*-methyl­pyrrole-2-carbaldehyde and 3-amino-1,2,4-triazole, the C=N double bond linking the two aromatic rings has a *Z* conformation. The two rings are twisted by 24.20 (5)°. A chain motif results from N—H⋯N hydrogen bonding.

## Related literature

For a related structure, see: Arfan *et al.* (2008 ▶).
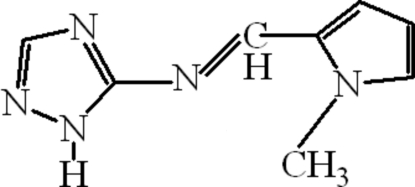


## Experimental

### 

#### Crystal data

C_8_H_9_N_5_
*M* *_r_* = 175.20
Monoclinic, 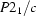

*a* = 7.2519 (3) Å
*b* = 12.8616 (6) Å
*c* = 9.7445 (4) Å
β = 101.917 (2)°
*V* = 889.29 (7) Å^3^
*Z* = 4
Mo *K*α radiation
μ = 0.09 mm^−1^
*T* = 296 (2) K
0.26 × 0.20 × 0.16 mm

#### Data collection

Bruker Kappa APEXII CCD diffractometer
Absorption correction: multi-scan (*SADABS*; Bruker, 2005 ▶) *T*_min_ = 0.976, *T*_max_ = 0.988
10203 measured reflections
2212 independent reflections
1651 reflections with *I* > 2σ(*I*)
*R*_int_ = 0.024

#### Refinement

*R*[*F* ^2^ > 2σ(*F* ^2^)] = 0.037
*wR*(*F* ^2^) = 0.107
*S* = 1.03
2212 reflections
136 parameters
H atoms treated by a mixture of independent and constrained refinement
Δρ_max_ = 0.16 e Å^−3^
Δρ_min_ = −0.17 e Å^−3^

### 

Data collection: *APEX2* (Bruker, 2007 ▶); cell refinement: *SAINT* (Bruker, 2007 ▶); data reduction: *SAINT*; program(s) used to solve structure: *SHELXS97* (Sheldrick, 2008 ▶); program(s) used to refine structure: *SHELXL97* (Sheldrick, 2008 ▶); molecular graphics: *ORTEP-3 for Windows* (Farrugia, 1997 ▶) and *PLATON* (Spek, 2003 ▶); software used to prepare material for publication: *WinGX* (Farrugia, 1999 ▶) and *PLATON*.

## Supplementary Material

Crystal structure: contains datablocks global, I. DOI: 10.1107/S160053680804097X/ng2523sup1.cif

Structure factors: contains datablocks I. DOI: 10.1107/S160053680804097X/ng2523Isup2.hkl

Additional supplementary materials:  crystallographic information; 3D view; checkCIF report

## Figures and Tables

**Table 1:** Hydrogen-bond geometry (Å, °)

*D*—H⋯*A*	*D*—H	H⋯*A*	*D*⋯*A*	*D*—H⋯*A*
N3—H3*n*⋯N5^i^	0.91 (1)	1.92 (1)	2.8225 (12)	171 (1)

Symmetry code: (i) 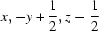.

**Fig. 1.**
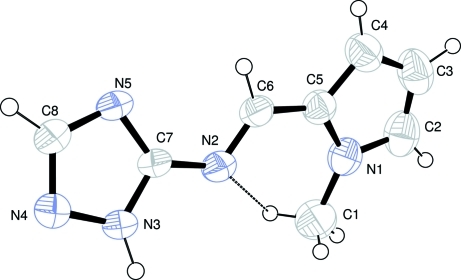
ORTEP-3 for Windows (Farrugia, 1997) drawing of the title compound, C8H9N5, with the atom numbering scheme. The thermal ellipsoids are drawn at the 50% probability level. H-atoms are shown by small circles of arbitrary radii. The intramolecular H-bonding is shown by dashed lines.

**Fig. 2.**
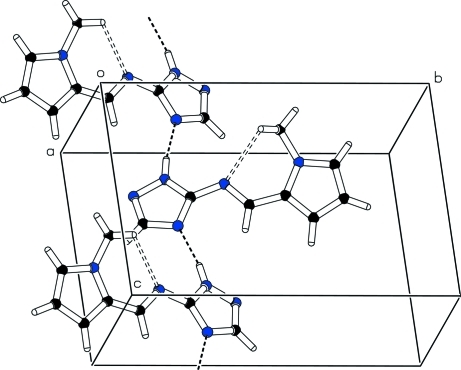
The partial unit cell packing of (I) (Spek, 2003) showing the interamolecular and intermolecular hydrogen bonding showing that polymeric sheets are formed.

## supplementary crystallographic information

### Comment

1,2,4-triazole ring is a basic aromatic ring and it possess various medicinal properties. The title compound (I), has been prepared to utilize it as an intermediate ligand and for complexation with various metals.

In the crystal structure of (I), (Fig 1), the pyrrole ring A(N1,C2—C5) is connected to the 1,2,4 triazole ring B(C7/N3/N4/C8/N5) through the Shiff bond C==N. There exist an intramolecular and an intermolecular H-bond (Fig 2), Table 1. The bond distances and bond angles of ring B are compareable as observed in the same moiety of 3-(2-Benzamidophenyl)-4-(4-hydroxyphenyl)- 5-methyl-4*H*-1,2,4-triazol-1-ium chloride (Arfan *et al.*, 2008). Due to intermolecular H-bonding, the compound forms polymeric sheets. The dihedral angle between the rings A and B is 24.20 (5)°. The molecules are stabilized due to π-π interactions between the centroids CgA and CgB of rings A and B respectively. The centroid to centroid, CgA···CgB^i^ [Symmetry code: i = 1 - *x*, 1 - *y*, 1 - *z*] and CgB···CgA^ii^ [Symmetry code: ii = - *x*, 1 - *y*, 1 - *z*] is 3.9008 (8) and 3.9009 (8) Å, respectively.

### Experimental

*N*-methyl pyrrole-2-carboxyaldehyde (1.047 ml, 0.01 *M*) in methanol solution (10 ml) was added to magnetically stirred methanol solution (20 ml) of 3-amino 1,2,4 triazole (0.84 g m, 0.01 *M*) and mixture refluxed for 5 h through monitoring by TLC. After completion of the reaction, the resultant mixture was cooled to room temperature, filtered and reduced nearly half of its volume by rotary. It was then allowed to stay at room temperature for 2 days which resulted in the formation of a colorless solid product. It was filtered, washed with methanol and recrystallized with a mixture of ethanol:methanol (1:1).

### Refinement

H-atoms were positioned geometrically, with C—H = 0.96 Å for methyl carbon and constrained to ride on their parent atom. The coordinates of all other H-atoms were refined. The *U*_iso_(H) = *xU*_eq_(C, N), where *x* = 1.5 for methyl H and *x* = 1.2 for all other H atoms.

### Crystal data

**Table:**

C_8_H_9_N_5_	*F*(000) = 368
*M**_r_* = 175.20	*D*_x_ = 1.309 Mg m^−^^3^
Monoclinic, *P*2_1_/*c*	Mo *K*α radiation, λ = 0.71073 Å
Hall symbol: -P 2ybc	Cell parameters from 1859 reflections
*a* = 7.2519 (3) Å	θ = 2.7–28.3°
*b* = 12.8616 (6) Å	µ = 0.09 mm^−^^1^
*c* = 9.7445 (4) Å	*T* = 296 K
β = 101.917 (2)°	Prismatic, orange
*V* = 889.29 (7) Å^3^	0.26 × 0.20 × 0.16 mm
*Z* = 4	

### Data collection

**Table:**

Bruker Kappa APEXII CCD diffractometer	2212 independent reflections
Radiation source: fine-focus sealed tube	1651 reflections with *I* > 2σ(*I*)
graphite	*R*_int_ = 0.024
Detector resolution: 7.4 pixels mm^-1^	θ_max_ = 28.3°, θ_min_ = 2.7°
ω scans	*h* = −5→9
Absorption correction: multi-scan (*SADABS*; Bruker, 2005)	*k* = −16→17
*T*_min_ = 0.976, *T*_max_ = 0.988	*l* = −12→12
10203 measured reflections	

### Refinement

**Table:**

Refinement on *F*^2^	Primary atom site location: structure-invariant direct methods
Least-squares matrix: full	Secondary atom site location: difference Fourier map
*R*[*F*^2^ > 2σ(*F*^2^)] = 0.037	Hydrogen site location: inferred from neighbouring sites
*wR*(*F*^2^) = 0.107	H atoms treated by a mixture of independent and constrained refinement
*S* = 1.03	*w* = 1/[σ^2^(*F*_o_^2^) + (0.0556*P*)^2^ + 0.079*P*] where *P* = (*F*_o_^2^ + 2*F*_c_^2^)/3
2212 reflections	(Δ/σ)_max_ = 0.001
136 parameters	Δρ_max_ = 0.16 e Å^−^^3^
0 restraints	Δρ_min_ = −0.17 e Å^−^^3^

### Special details

**Table:**

Geometry. Bond distances, angles *etc*. have been calculated using the rounded fractional coordinates. All su's are estimated from the variances of the (full) variance-covariance matrix. The cell e.s.d.'s are taken into account in the estimation of distances, angles and torsion angles
Refinement. Refinement of *F*^2^ against ALL reflections. The weighted *R*-factor *wR* and goodness of fit *S* are based on *F*^2^, conventional *R*-factors *R* are based on *F*, with *F* set to zero for negative *F*^2^. The threshold expression of *F*^2^ > σ(*F*^2^) is used only for calculating *R*-factors(gt) *etc*. and is not relevant to the choice of reflections for refinement. *R*-factors based on *F*^2^ are statistically about twice as large as those based on *F*, and *R*- factors based on ALL data will be even larger.

### Fractional atomic coordinates and isotropic or equivalent isotropic displacement parameters (Å^2^)

**Table:**

	*x*	*y*	*z*	*U*_iso_*/*U*_eq_	
N1	0.11672 (13)	0.58683 (8)	0.33358 (11)	0.0543 (3)	
N2	0.27287 (13)	0.37308 (7)	0.38528 (9)	0.0459 (3)	
N3	0.38353 (14)	0.21080 (7)	0.32861 (9)	0.0475 (3)	
N4	0.44733 (16)	0.11865 (8)	0.38789 (10)	0.0552 (3)	
N5	0.37569 (15)	0.22874 (7)	0.54902 (9)	0.0494 (3)	
C1	0.0572 (2)	0.53330 (12)	0.20067 (14)	0.0754 (5)	
C2	0.0834 (2)	0.68853 (11)	0.35562 (19)	0.0691 (5)	
C3	0.1530 (2)	0.71296 (12)	0.49152 (19)	0.0719 (6)	
C4	0.23118 (19)	0.62366 (11)	0.55771 (16)	0.0609 (5)	
C5	0.20967 (15)	0.54493 (9)	0.45902 (12)	0.0471 (3)	
C6	0.27772 (15)	0.44120 (9)	0.48170 (12)	0.0451 (3)	
C7	0.34307 (15)	0.27546 (8)	0.42465 (10)	0.0415 (3)	
C8	0.43865 (19)	0.13408 (10)	0.51969 (12)	0.0545 (4)	
H1A	−0.03220	0.57556	0.13837	0.1132*	
H1B	−0.00032	0.46826	0.21599	0.1132*	
H1C	0.16470	0.52068	0.15976	0.1132*	
H2	0.025 (2)	0.7315 (13)	0.2805 (17)	0.0829*	
H3	0.149 (2)	0.7801 (13)	0.5321 (17)	0.0862*	
H3N	0.3769 (18)	0.2235 (10)	0.2359 (14)	0.0571*	
H4	0.294 (2)	0.6150 (11)	0.6550 (17)	0.0730*	
H6	0.3326 (17)	0.4259 (9)	0.5798 (14)	0.0541*	
H8	0.4755 (19)	0.0815 (11)	0.5904 (15)	0.0655*	

### Atomic displacement parameters (Å^2^)

**Table:**

	*U*^11^	*U*^22^	*U*^33^	*U*^12^	*U*^13^	*U*^23^
N1	0.0532 (5)	0.0502 (6)	0.0587 (6)	0.0034 (4)	0.0099 (4)	0.0071 (5)
N2	0.0558 (5)	0.0467 (5)	0.0355 (5)	0.0026 (4)	0.0102 (4)	0.0039 (4)
N3	0.0696 (6)	0.0446 (5)	0.0290 (4)	−0.0016 (4)	0.0116 (4)	−0.0003 (4)
N4	0.0813 (7)	0.0435 (6)	0.0415 (5)	0.0025 (5)	0.0143 (5)	−0.0004 (4)
N5	0.0708 (6)	0.0486 (5)	0.0302 (4)	0.0041 (4)	0.0137 (4)	0.0046 (4)
C1	0.0920 (10)	0.0713 (10)	0.0546 (8)	0.0084 (8)	−0.0044 (7)	0.0100 (7)
C2	0.0635 (8)	0.0525 (8)	0.0924 (11)	0.0090 (6)	0.0187 (7)	0.0113 (7)
C3	0.0720 (9)	0.0503 (8)	0.0983 (12)	0.0023 (7)	0.0292 (8)	−0.0106 (8)
C4	0.0618 (8)	0.0581 (8)	0.0653 (8)	−0.0038 (6)	0.0189 (6)	−0.0101 (6)
C5	0.0455 (5)	0.0481 (6)	0.0494 (6)	−0.0023 (5)	0.0135 (4)	0.0009 (5)
C6	0.0499 (6)	0.0485 (6)	0.0373 (5)	−0.0020 (5)	0.0102 (4)	0.0035 (5)
C7	0.0515 (6)	0.0444 (6)	0.0288 (5)	−0.0021 (4)	0.0090 (4)	0.0009 (4)
C8	0.0782 (8)	0.0466 (7)	0.0388 (6)	0.0043 (6)	0.0120 (5)	0.0068 (5)

### Geometric parameters (Å, °)

**Table:**

N1—C1	1.4515 (17)	C3—C4	1.380 (2)
N1—C2	1.3553 (18)	C4—C5	1.3830 (19)
N1—C5	1.3779 (15)	C5—C6	1.4238 (16)
N2—C6	1.2798 (14)	C1—H1A	0.9600
N2—C7	1.3792 (14)	C1—H1B	0.9600
N3—N4	1.3572 (14)	C1—H1C	0.9600
N3—C7	1.3293 (14)	C2—H2	0.945 (16)
N4—C8	1.3139 (15)	C3—H3	0.953 (17)
N5—C7	1.3294 (13)	C4—H4	0.970 (16)
N5—C8	1.3514 (16)	C6—H6	0.977 (13)
N3—H3N	0.910 (13)	C8—H8	0.963 (14)
C2—C3	1.353 (3)		
			
N1···N2	2.9763 (14)	C1···H3^vii^	3.058 (16)
N2···N1	2.9763 (14)	C6···H1B	2.9600
N2···C1	2.9609 (17)	C7···H2^iv^	3.039 (16)
N3···N5^i^	2.8225 (12)	C7···H3N^iii^	2.992 (13)
N3···N5	2.1725 (12)	C8···H8^ii^	3.083 (14)
N4···C8^ii^	3.4283 (17)	C8···H3N^iii^	2.896 (13)
N4···N5	2.2532 (14)	H1A···H2	2.4200
N5···N3^iii^	2.8225 (12)	H1B···N2	2.6100
N5···N4	2.2532 (14)	H1B···C6	2.9600
N2···H1B	2.6100	H1C···N2	2.8900
N2···H1C	2.8900	H2···H1A	2.4200
N3···H2^iv^	2.946 (15)	H2···N3^viii^	2.946 (15)
N4···H8^ii^	2.634 (14)	H2···C7^viii^	3.039 (16)
N5···H6	2.580 (12)	H3···C1^ix^	3.058 (16)
N5···H3N^iii^	1.920 (13)	H3N···N5^i^	1.920 (13)
C1···N2	2.9609 (17)	H3N···C7^i^	2.992 (13)
C3···C7^v^	3.5793 (19)	H3N···C8^i^	2.896 (13)
C3···C8^v^	3.576 (2)	H3N···H6^i^	2.431 (18)
C4···C7^v^	3.3206 (18)	H4···H6	2.572 (19)
C5···C5^vi^	3.4963 (16)	H6···N5	2.580 (12)
C6···C6^v^	3.5119 (16)	H6···H4	2.572 (19)
C7···C3^v^	3.5793 (19)	H6···H3N^iii^	2.431 (18)
C7···C4^v^	3.3206 (18)	H8···N4^ii^	2.634 (14)
C8···C3^v^	3.576 (2)	H8···C8^ii^	3.083 (14)
C8···N4^ii^	3.4283 (17)		
			
C1—N1—C2	124.70 (12)	N3—C7—N5	109.60 (9)
C1—N1—C5	127.37 (11)	N4—C8—N5	115.42 (11)
C2—N1—C5	107.91 (11)	N1—C1—H1A	109.00
C6—N2—C7	117.80 (9)	N1—C1—H1B	109.00
N4—N3—C7	110.62 (8)	N1—C1—H1C	109.00
N3—N4—C8	101.73 (10)	H1A—C1—H1B	109.00
C7—N5—C8	102.63 (9)	H1A—C1—H1C	109.00
N4—N3—H3N	121.5 (8)	H1B—C1—H1C	109.00
C7—N3—H3N	127.8 (8)	N1—C2—H2	120.5 (10)
N1—C2—C3	109.66 (14)	C3—C2—H2	129.7 (10)
C2—C3—C4	107.35 (14)	C2—C3—H3	125.4 (10)
C3—C4—C5	107.93 (13)	C4—C3—H3	127.2 (10)
N1—C5—C4	107.15 (11)	C3—C4—H4	128.1 (9)
C4—C5—C6	126.33 (11)	C5—C4—H4	124.0 (9)
N1—C5—C6	126.48 (10)	N2—C6—H6	121.6 (7)
N2—C6—C5	124.85 (11)	C5—C6—H6	113.5 (7)
N2—C7—N3	119.75 (9)	N4—C8—H8	122.1 (9)
N2—C7—N5	130.62 (9)	N5—C8—H8	122.5 (9)
			
C1—N1—C5—C4	−177.86 (12)	C7—N3—N4—C8	0.72 (13)
C1—N1—C2—C3	178.37 (12)	N3—N4—C8—N5	−0.41 (15)
C5—N1—C2—C3	0.21 (16)	C8—N5—C7—N3	0.52 (13)
C2—N1—C5—C6	−177.44 (12)	C8—N5—C7—N2	178.36 (12)
C2—N1—C5—C4	0.23 (14)	C7—N5—C8—N4	−0.06 (15)
C1—N1—C5—C6	4.47 (19)	N1—C2—C3—C4	−0.57 (17)
C6—N2—C7—N5	18.65 (18)	C2—C3—C4—C5	0.70 (17)
C6—N2—C7—N3	−163.70 (11)	C3—C4—C5—C6	177.10 (12)
C7—N2—C6—C5	−179.40 (11)	C3—C4—C5—N1	−0.57 (15)
N4—N3—C7—N5	−0.82 (14)	C4—C5—C6—N2	−172.36 (12)
N4—N3—C7—N2	−178.93 (10)	N1—C5—C6—N2	4.88 (19)

Symmetry codes: (i) *x*, −*y*+1/2, *z*−1/2; (ii) −*x*+1, −*y*, −*z*+1; (iii) *x*, −*y*+1/2, *z*+1/2; (iv) −*x*, *y*−1/2, −*z*+1/2; (v) −*x*+1, −*y*+1, −*z*+1; (vi) −*x*, −*y*+1, −*z*+1; (vii) *x*, −*y*+3/2, *z*−1/2; (viii) −*x*, *y*+1/2, −*z*+1/2; (ix) *x*, −*y*+3/2, *z*+1/2.

### Hydrogen-bond geometry (Å, °)

**Table:**

*D*—H···*A*	*D*—H	H···*A*	*D*···*A*	*D*—H···*A*
N3—H3n···N5^i^	0.910 (13)	1.920 (13)	2.8225 (12)	171.3 (12)
C1—H1B···N2	0.9600	2.6100	2.9609 (17)	102.00

Symmetry codes: (i) *x*, −*y*+1/2, *z*−1/2.
